# Charting the future of esophageal cancer translation: insights from clinical trial landscape

**DOI:** 10.1097/JS9.0000000000002254

**Published:** 2025-01-28

**Authors:** Hujian Hong, Yijiang He, Yan Li, Yongyan Shen, Yanli Qu

**Affiliations:** a Department of Radiotherapy, Cancer Hospital of China Medical University, Liaoning Cancer Hospital & Institute, Cancer Hospital of Dalian University of Technology, Shenyang, China; b Graduate School, Dalian Medical University, Dalian, China; c Graduate School, China Medical University, Shenyang, China

*To the Editor:*

Esophageal cancer continues to pose a substantial global health challenge, characterized by its high incidence and mortality rates. As a pivotal focus within oncological research, the quest for more efficacious treatment modalities and the advancement of novel therapeutic approaches are of paramount importance^[1]^. Conventional treatments, encompassing surgical resection, chemotherapy, radiotherapy, and various adjunct therapies, have yielded only modest improvements in patient survival rates^[2,3]^. Nonetheless, their efficacy is notably constrained in the context of advanced-stage esophageal cancer, where the prognosis remains poor^[4,5]^. This has led to focused efforts on evolving targeted and immunotherapeutic therapies, which are vital in the current medical landscape. In particular, recent significant studies such as the ESCORT-NEO/NCCES01 trial have shown promising results by integrating camrelizumab with neoadjuvant chemotherapy, offering higher pathological complete response rates and acceptable safety profiles^[6]^. Similarly, the BRES-1 study demonstrated significant improvements in R0 resection rates with the use of carrelizumab combined with chemotherapy, marking a substantial advancement in the treatment of esophageal cancer^[7]^. The CAP 02 Re-challenge trial introduced the efficacy and safety of camrelizumab combined with apatinib for patients with advanced esophageal squamous cell carcinoma who had previously received treatment with immune checkpoint inhibitors (ICIs)^[8]^. The Keystone-001 trial is a prospective study on the neoadjuvant treatment of resectable esophageal squamous cell carcinoma with pembrolizumab combined with chemotherapy^[9]^. These developments highlight the ongoing commitment of many institutions and researchers to innovate within the realm of immunotherapy, bringing forth a wave of clinical trials that pave the way for enhanced therapeutic strategies. The main objective of this study is to systematically analyze the trends of clinical trials in the treatment of esophageal cancer in recent years, with a particular focus on new advances in immunotherapy and precision medicine.

This study utilizes the INFORMA database (https://pharma.id.informa.com/) to systematically analyze the clinical trial landscape for esophageal cancer. Employing professional medical subject headings (MeSH), we retrieved terms including “Disease: esophageal cancer” and got a total of 2966 clinical trials (flowchart). Furthermore, the research delves into various phases of clinical trials, the types of drugs used, and how these drugs compare to conventional treatment methods. Through this approach, we provide a comprehensive overview of the latest advancements and future directions in the field of esophageal cancer treatment. Although the INFORMA database provides extensive information on clinical trials, its possible limitations include geographic bias and the absence of certain small trials, which may affect the comprehensiveness of study results.

This article presents a comprehensive analysis of the contemporary landscape and emerging trends in global clinical trials for esophageal cancer, revealing several critical findings. Since 2010, there has been a significant increase in the number of clinical trials focusing on esophageal cancer, particularly in Phases I and II, suggesting a gradual advancement of new treatments into the clinical validation stage. As illustrated in Figure 1A, the increase in the number of Phases I and II trials underscores a vigorous effort by researchers in the preliminary investigation of innovative therapies.

**Figure 1.**
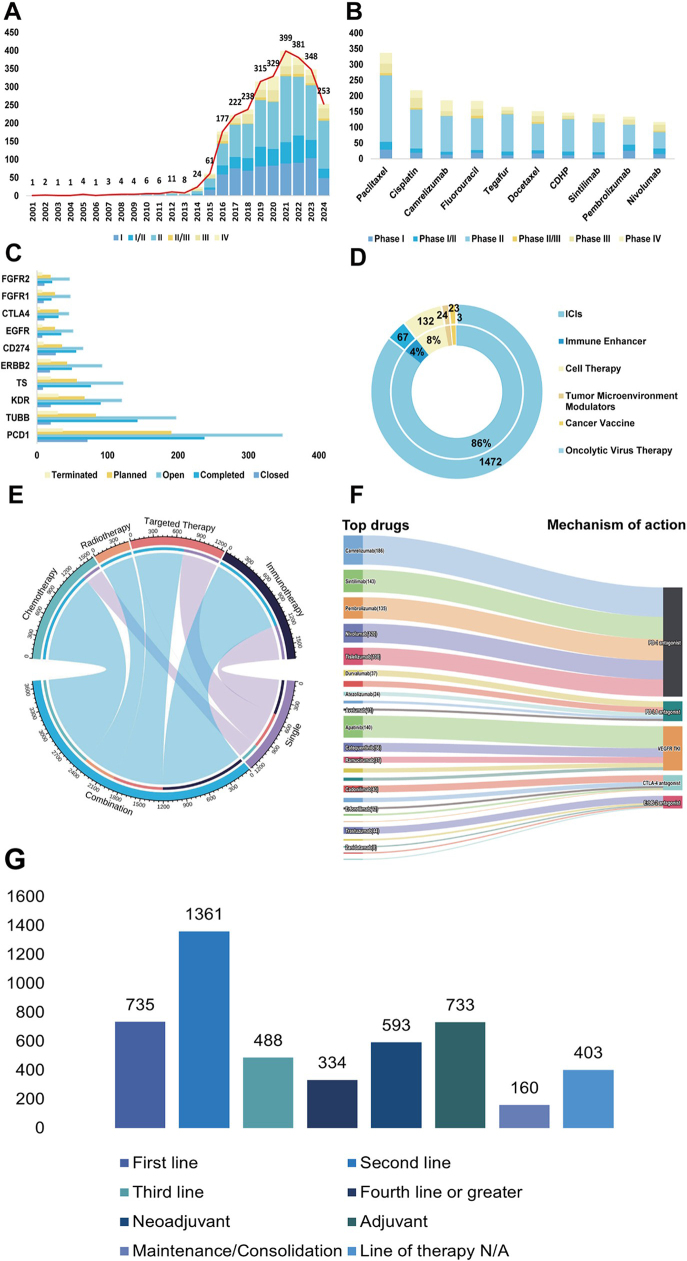
Analysis and trends in esophageal cancer clinical trials. (A) Annual distribution of esophageal cancer clinical trials by phase from 2001 to 2024. (B) Distribution of various drug types in esophageal cancer clinical trials. (C) Distribution of different targets in esophageal cancer clinical trials and their trial statuses. (D) Distribution of various immunotherapy applications in esophageal cancer clinical trials. (E) Comparative application of single and combination therapies in esophageal cancer clinical trials. (F) Top drugs and mechanisms of action. (G) Line of therapy.

**Figure 1A.**
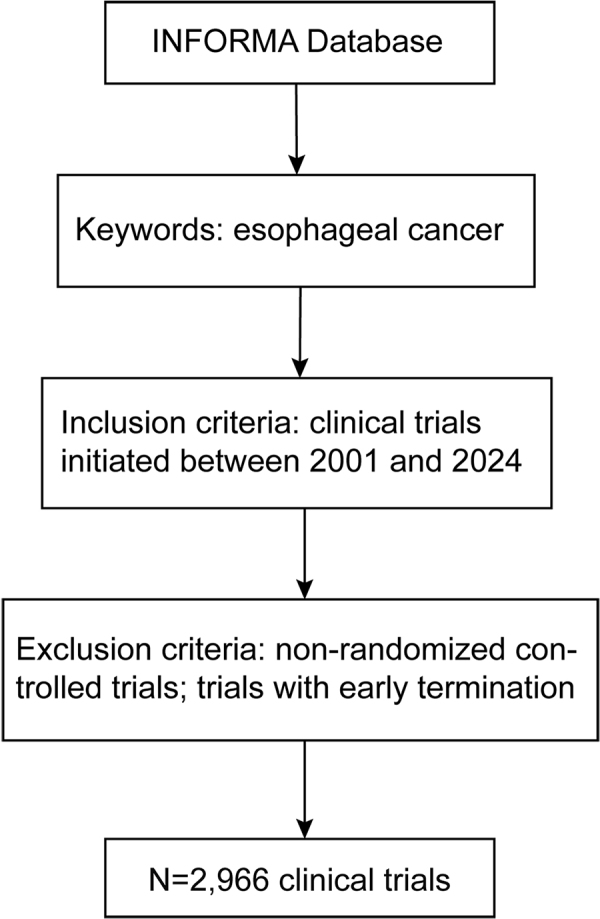
Flowchart.

The analysis of drug types, as shown in Figure 1B, reveals that traditional chemotherapy agents such as cisplatin and docetaxel continue to be widely utilized. Concurrently, emerging targeted and immunotherapy drugs, such as pembrolizumab, have secured a significant presence across various trial phases. The current state of clinical trials, illustrated in Figure 1C, shows diversity in drug targets like PCD1, TUBB, and KDR, with some trials either completed or advanced to later stages. Whereas PDC1 is a key target of ICIs, which activates T-cells to attack cancer cells by blocking the interaction of PD-1 with its ligand PD-L1. TUBB and KDR play an important role in cytoskeleton stabilization and angiogenesis, respectively, which are new targets being explored in clinical trials, and are precisely the new targets currently being explored in clinical trials. Figure 1D focuses on the predominance of ICIs within the scope of immunotherapies, accentuating their transformative impact on the treatment modalities for esophageal cancer. This insight is complemented by the analysis in Figure 1E, which contrasts the use of immunotherapy and targeted therapy, particularly in combination regimens, highlighting their potential to significantly enhance treatment outcomes. Furthermore, Figure 1F showcases the most extensively studied drugs and their mechanisms of action, with a pronounced focus on PD-1 and PD-L1 antagonists as the leading therapeutic strategies currently under investigation. Figure 1G illustrates the distribution of patients across various therapeutic lines for esophageal cancer, emphasizing the application of neoadjuvant and adjuvant treatments. Predominantly, the largest number of patients is treated with first-line therapies, followed by adjuvant and second-line treatments, highlighting the reliance on and prioritization of these strategies in the management of esophageal cancer. This observation highlights the critical role of these inhibitors in propelling forward the therapeutic approaches against esophageal cancer. Meanwhile Table 1 demonstrates the main features of current clinical trials in esophageal cancer.

**Table 1:** The key features of current esophageal cancer clinical trials

Characteristics and trial type	China, *N*	USA, *N*	Others, *N*	Monotherapy, *N*	Combination therapy, *N*	Total *N*
Phase I	418	296	82	539	257	796
Phase I/II	192	192	41	230	195	425
Phase II	824	155	245	331	893	1224
Phase II/III	29	6	12	16	31	47
Phase III	187	45	3	77	158	235
Phase IV	167	5	45	78	139	217
Trial status						
Closed	89	108	20	91	126	217
Completed	440	218	179	381	456	837
Open	805	247	109	510	651	1161
Planned	395	15	77	160	327	487
Terminated	96	114	50	136	124	260
Primary endpoint						
Safety/toxicity	34	75	16	79	46	125
Efficacy	58	35	23	43	73	116

Patients diagnosed with early-stage esophageal cancer typically achieve favorable prognoses through surgical intervention. However, those diagnosed at stages where surgery is not viable require comprehensive therapies such as radiation or pharmacotherapy. Chemoimmunotherapy, a combination of chemotherapy and immunotherapy, has profoundly transformed the treatment paradigm for advanced metastatic esophageal cancer, establishing itself as the standard first-line therapy for late-stage esophageal squamous cell carcinoma, demonstrating significant efficacy. Currently, ICIs are central to esophageal cancer immunotherapy. Their safety is widely acknowledged, and they have been approved by the Food and Drug Administration and European Medicines Agency as primary and secondary treatment options, including tislelizumab, pembrolizumab, nivolumab, and ipilimumab. The NMPA has also approved a roster of six immunotherapeutic drugs: tislelizumab, pembrolizumab, nivolumab, toripalimab, camrelizumab, and sintilimab.

The Chinese government’s recent endorsement of the “Comprehensive Support for Innovative Drug Development Strategy” aims to strengthen the national pharmaceutical industry and significantly impact the development of treatments for esophageal cancer, particularly immunotherapies. This plan is expected to accelerate clinical trials of new therapies by streamlining approval processes and enhancing medical institution evaluation mechanisms, promoting the rapid integration of advanced treatment methodologies into clinical practice and enhancing research into novel immunotherapeutic drugs and their combinations.

Based on the current narrative surrounding clinical trials for esophageal cancer, the prospects for precision immunotherapy are increasingly promising. By integrating innovative targeted treatments such as antiangiogenic agents and anti-EGFR drugs with radiotherapy, the additive approach of immunotherapy significantly enhances therapeutic efficacy, while subtractive strategies reduce side effects while preserving effectiveness. Precision immunotherapy clinical trials, like the SKYSCRAPER series, have validated the efficacy of combining TIGIT inhibitors with PD-L1 inhibitors, substantially prolonging patient survival^[10]^. Furthermore, the combination of pembrolizumab, nivolumab, and sintilimab with chemotherapy, as well as dual immunotherapy regimens (Opdivo + Yervoy), has significantly extended both overall and progression-free survival for patients with esophageal cancer^[11-14]^. Based on the current study, we recommend that PD-1 inhibitors be considered as a first-line treatment option in the management of esophageal cancer, especially for patients with advanced disease. Further studies should explore biomarkers of response to such treatment in different patients.

These therapeutic advances provide diverse treatment options for patients with advanced esophageal cancer, marking a shift toward a more personalized and effective treatment strategy driven by immunotherapy in combination with other treatment modalities. This comprehensive and targeted approach is expected to further improve patient survival and quality of life, bringing hope for a breakthrough in the treatment of esophageal cancer. Future research needs to focus on the effects of combining immunotherapy with conventional treatments, particularly how these strategies can be optimized in diverse patient populations to improve treatment outcomes and patient survival. Key research questions include the development of immunotherapeutic strategies targeting biomarkers specific to esophageal cancer and how to overcome the resistance problems observed with current treatments. In addition, investigating how to integrate multiple treatment modalities to improve survival and quality of life for patients with advanced esophageal cancer is a major challenge for the field.

## Data Availability

The datasets generated and analyzed during the current study are available in the INFORMA database (https://pharma.id.informa.com/).

## Research registration unique identifying number (UIN)

The INFORMA database (https://pharma.id.informa.com/)
